# Outcomes of Bariatric Surgery in Morbidly Obese Patients with Multiple Sclerosis

**DOI:** 10.1155/2017/1935204

**Published:** 2017-02-19

**Authors:** Kalman Bencsath, Adham Jammoul, Ali Aminian, Hideharu Shimizu, Carolyn J. Fisher, Philip R. Schauer, Alexander Rae-Grant, Stacy A. Brethauer

**Affiliations:** ^1^Bariatric and Metabolic Institute, Cleveland Clinic, Cleveland, OH, USA; ^2^Department of General Surgery, Cleveland Clinic, Cleveland, OH, USA; ^3^Department of Neurology, Cleveland Clinic, Cleveland, OH, USA; ^4^Mellen Center for Multiple Sclerosis, Neurological Institute, Cleveland Clinic, Cleveland, OH, USA

## Abstract

Obesity is common in patients with multiple sclerosis (MS); however, safety and efficacy of bariatric surgery in this population remain unclear. A database of 2,918 was retrospectively reviewed, yielding 22 (0.75%) severely obese patients with MS who underwent bariatric surgery. Sixteen surgical patients with complete follow-up data were matched to a nonsurgical control group of MS patients, based on age, BMI, MS subtype, and length of follow-up. MS relapse rates and trends in the timed twenty-five foot walk test (T25FW) were compared. In the surgical group (gastric bypass *n* = 19, sleeve gastrectomy *n* = 3), preoperative BMI was 46.5 ± 7.2 Kg/m^2^ and average excess weight was 60.4 kg. Follow-up data was collected at 59.0 ± 29.8 months. There were two major and four minor complications. Five patients required readmission and there were no mortalities. Percent excess weight loss was 75.5 ± 27.0%. In the 16 patients with follow-up data, patients who underwent bariatric surgery were significantly faster on the T25FW compared to the nonsurgical population. In conclusion, bariatric surgery is relatively safe and effective in achieving weight loss in patients with MS. In addition, surgery may help patients maintain ambulation. Findings support the need for further studies on bariatric surgery and disease-specific outcomes in this population.

## 1. Introduction

Since 1999, obesity in the United States has been trending upwards, with the prevalence of obesity increasing from 27% to 35% in adult men and 33 to 36% in adult women [1–3]. Multiple sclerosis (MS) patients are not immune to the obesity pandemic. While the true rate of obesity in patients with MS is uncertain, estimates range from 21.2%–32.7% [4, 5]. Accumulating evidence suggests that adolescent obesity is a risk factor for developing MS, particularly in the context of certain genetic predispositions [6, 7]. Moreover, MS commonly impairs walking ability, which can dramatically affect daily routines and quality of life [8], and for those patients with excess weight, it stands to reason that the disability from the disease may be even more pronounced. Despite the overlap between obesity and MS, literature pertaining to the safety and efficacy of bariatric surgery in the MS population is scarce. Therefore, we retrospectively reviewed our database and collected outcome data on bariatric surgery in this patient population. In addition, we matched these individuals to nonsurgical patients from our Neurologic Institute to compare walking outcomes between the two groups. To our knowledge, this is the first retrospective case-control series evaluating the long-term surgical and ambulatory outcomes of bariatric surgery in MS patients.

## 2. Methods

Approval for this project was obtained from our Institutional Review Board. A retrospective review of our bariatric surgical database from 2004 to 2012 was performed. A search by diagnosis and ICD-9 code corresponding to MS was used to identify the subset of individuals who carried the diagnosis of MS at the time of surgery. Demographic data, including age at the time of surgery, gender, preoperative weight and body mass index (BMI), and postoperative weight and BMI, were collected. Additional surgical outcomes analyzed included length of hospital stay, adverse events, readmissions, and mortality.

A control group of morbidly obese patients with MS who did not undergo bariatric surgery was identified through chart review of all MS patients presenting to our MS center during a consecutive 2-month period from February 1st to March 31st 2012. This group, within the limitations of a case-control series, was matched on BMI, age, MS subtype, and time interval between serial neurologic testing. The timed twenty-five foot walk test (T25FW), the European Quality of Life-5 Dimensions (EQ-5D), and the Patient Health Questionnaire (PHQ-9) were collected and analyzed whenever available. T25FW variability, defined as [(T25FW_(longest)_/T25FW_(shortest)_) − 1] × 100, was determined for each patient with available data. A cutoff of 20% or greater T25FW variability was considered reflective of significant clinical change, as validated in prior studies [9–11]. In addition, for each patient in either group, the preoperative (or initial) T25FW was subtracted from the postoperative (or most recent) T25FW (i.e., Post-Pre). This is designated herein as ΔT25FW. Fisher's exact test and *t*-test were utilized to analyze the significance between groups. Data were analyzed using SPSS (Version 21, Chicago Inc., IL, USA) and a *p* < 0.05 was deemed statistically significant.

## 3. Results

Review of the database yielded 2,918 patients who underwent bariatric procedures during the study period. Twenty-two of these patients (0.75%) had a diagnosis of MS prior to bariatric surgery. Twenty patients were female and two were male with a mean age of 45.7 ± 9.6 years (range 32–62 years). The mean BMI was 46.5 ± 7.2 Kg/m^2^ (range 35.1–64.3 Kg/m^2^).

Nineteen patients underwent laparoscopic Roux-en-Y gastric bypass (RYGB) and three patients underwent laparoscopic sleeve gastrectomy. One of the patients previously had a laparoscopic adjustable gastric band placed and subsequently underwent conversion to a RYGB. The mean length of hospital stay was 4.2 ± 4.4 days (range 2–23 days). One patient required a hospital stay of 23 days due to complications of an anastomotic leak at the jejunojejunostomy. Five patients required early readmission: one patient was readmitted for dehydration, one for a port site infection, one for an anastomotic ulcer, and one for a stricture managed with balloon dilation. One patient required reoperation two weeks following RYGB for an adhesive obstruction at the distal ileum related to a prior hysterectomy.

At last follow-up (mean = 59.0 ± 29.8 months) the mean percentage of excess weight loss was 75.5%  ±  27.0, and percentage of total weight loss was 31.5%  ±  11.8.

No patients were on corticosteroids for management of MS at the time of surgery. Five patients were receiving daily injections of glatiramir, an immune modulator, just prior to surgery, with one of these patients also receiving interferon beta-1a three times weekly. Four other patients were also receiving interferon beta-1a. Two patients were receiving weekly injections of natalizumab, a humanized monoclonal antibody directed against the cell adhesion molecule alpha4-integrin, for the treatment of MS.

The single patient with a port site infection was receiving interferon beta-1a and the patient with an anastomotic ulcer was receiving glatiramir injections pre-operatively, but was also taking meloxicam until two weeks before surgery. The patient whose course was complicated by a jejunojejunal anastomotic leak was not receiving immune modulators for MS. Recent use of immune modulators for treatment for MS was not associated with complications of any kind (*p* = 1.00).

**Figure 1:**
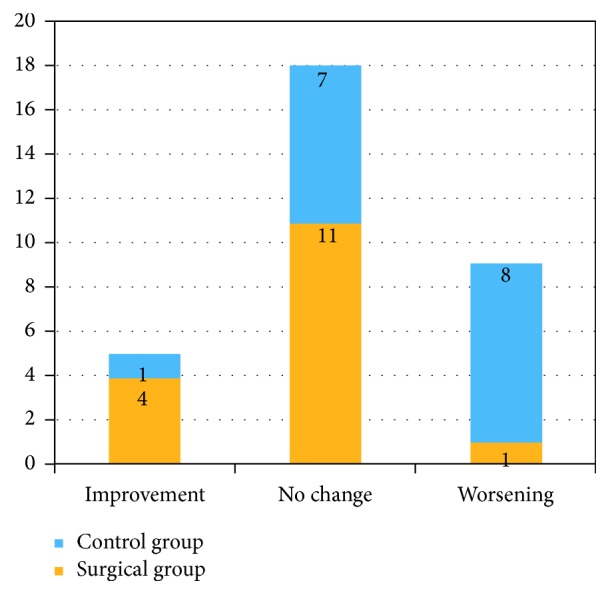
Distribution of T25FW variability across the two groups.

**Figure 2:**
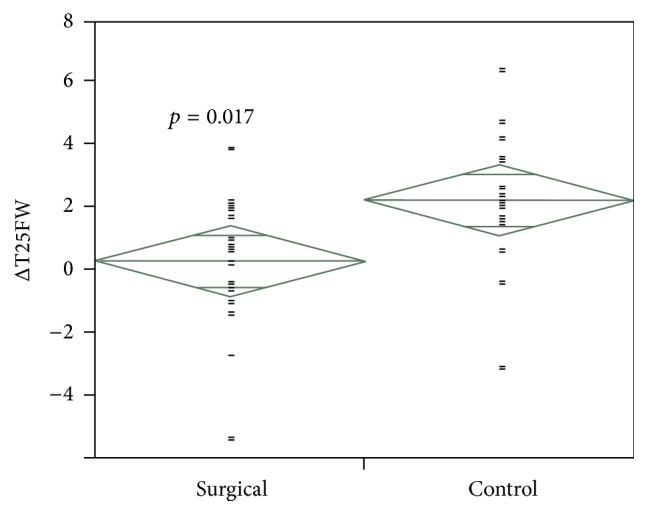
Distribution of (Post–Pre) T25FW differences between surgical and control groups.

Pre- and postoperative T25FW data were available for 16 of the 22 patients who had bariatric surgery; four patients did not seek care at our institution for their MS and two additional patients lacked serial T25FW measurements. The baseline characteristics of these 16 patients and the nonsurgical control group are shown in Table 1. The mean time frame between the two index visits was 41.2 months for the study sample and 39.8 months for the control group. For the surgical group, 11 patients (68.7%) showed no change in their walking speed, four patients (25%) exhibited improvement, and one patient experienced slowing. This is in contrast with the findings among the 16 patients comprising the control sample, in which 8 (50%) had slowing on serial testing, 7 (44%) showed no change, and one patient exhibited improvement (Figure 1). After calculating the ΔT25FW for each group, it was noted that, on average, MS patients who underwent bariatric surgery were walking 1.96 seconds faster than their nonsurgical cohort (*p* = 0.02; Figure 2).

As for the quality of life measures, EQ-5D and PHQ-9 scores had improved on repeat assessment in both groups, with a nonsignificant difference between the two samples (data not shown). In addition, it is worthwhile to note that the number of acute MS exacerbations during the follow-up period was 4 in the control group as compared to only 2 in the surgical group.

## 4. Discussion

In 1997, Flanagan reported the bariatric surgery outcomes in a heterogeneous case series of six patients with neurologic motor deficits, three of whom had MS. The mean excess weight loss was 77% at 18 months. Two of the MS patients demonstrated improved balance postoperatively while the third became wheelchair bound on long-term follow-up [12]. Lutrzykowski reported on a wheelchair-bound morbidly obese MS patient who experienced significant weight loss and improved mobility in her wheelchair after an open duodenal switch procedure [13].

Ambulation is frequently affected in MS patients, altering balance, impairing safety, and often slowing the speed of walking [14]. In many patients, ambulation is affected early in the disease, even when clinical measures of disability are minimally altered [15]. Of importance, patients with MS perceive difficulties with ambulation as a major issue for their health [16]. Ambulation limitations have an impact on activities, emotional status, quality of life, and health status [17]. A recent online survey revealed that two thirds of people with MS admitted to difficulty walking or maintaining balance, with most of them believing that that difficulty led to detrimental financial and social consequences such as unemployment [18].

No reports other than anecdotal data have been published on the surgical or ambulatory outcomes of bariatric surgery in MS patients. Our data are consistent with other studies reporting on bariatric surgery outcomes, particularly in higher risk populations, which have shown modest complication rates and a mean length of stay of 3-4 days [19, 20]. Long considered to be associated with an elevated risk of postoperative complications, the immune modulators used in this patient population do not seem to pose significant added risk. The readmission and overall complication rates are higher than typically seen and warrant further investigation and consideration of risk reduction measures in this patient population. A limitation of the study is the small sample size, however, and it is not known whether differences might be seen in larger samples. The average excess weight loss of 75.5% sustained by our study sample is on the upper end of the reported range of 57% to 77% seen in previous studies [19, 21].

With respect to MS outcome measures, our surgical patients were able to maintain stable walking speeds as measured by serial T25FW for the duration of follow-up, as compared to controls who were about 2 seconds slower on average. While this is an important finding, particularly in the context of limited research in this area, loss of excess weight is the most likely factor responsible for decreased walking time. This highlights a limitation of the current study, which was using this outcome measure in isolation. Future research should explore additional, well-accepted outcome measures of functional and disability status in this population following bariatric surgery.

The recent identification of childhood obesity as a risk factor for the development of MS raises the intriguing hypothesis that the metabolic effects of bariatric surgery may attenuate a unifying proinflammatory state underlying both obesity and MS [7, 22, 23]. Consistent with this idea, bariatric surgery has been found to have a significant positive effect on neurologic conditions such as pseudotumor cerebri, migraine, and Alzheimer's disease [24]. In this present study, there was no statistically significant difference in QOL measures between the two groups. The fact that worsening ambulation status in the control group did not translate into better QOL measures in the surgical cohort may be attributed to the small study sample size.

Due to growing evidence suggesting a link between obesity and MS, future research should seek to elucidate the nature of this relationship. Limitations of the current study pertain the cross-sectional and retrospective nature of the data analysis. In addition, while the control group was matched on relevant demographic and diagnostic variables, we do not have data on whether individuals in the control group were involved in nonsurgical weight management interventions, which may have attenuated our ability to detect differences in outcomes. Thus, a longitudinal controlled trial evaluating disease severity in patients with MS compared to MS patients undergoing bariatric surgery would help determine if bariatric surgery might be able to attenuate the disease and determine the mechanisms behind such a finding. Future studies should therefore be directed at disease-specific outcomes in bariatric surgery patients with MS.

Taken together, these data illustrate an overall acceptable safety profile of bariatric surgery in this patient population. In addition, we did not observe disease worsening or increased relapses following bariatric surgery. While the observational nature of this study precludes definitive conclusions, the results are encouraging. Our data suggest that bariatric surgery is a viable treatment option for the severely obese MS patient and that the surgical outcomes may be expected to be similar to the general bariatric population. The data is limited in the ability to confidently address the safety of immune modulators in the perioperative period, and this should be addressed on a case-by-case basis.

## 5. Conclusions

Bariatric surgery can be performed relatively safely and appears to be effective in patients with MS. The preoperative use of immune modulators for treatment of MS had no effect on perioperative complications in this small series. These data support that surgery can be offered as part of a comprehensive strategy for the treatment of severe obesity in this patient population. Any positive impacts of surgery on the management of symptoms relating to MS, such as ambulation, or upon the disease itself should be explored in future studies.

## Figures and Tables

**Table 1:** Baseline characteristics of the surgical and control groups.

	Surgical group (*n* = 16)	Control group (*n* = 16)
Age, y, mean (SD)	51 (10.5)	48.2 (12.2)
Gender F-M	15-1	13-3
BMI, kg/m^2^, mean (SD)	45 (4.7)	43.6 (3.7)
Type of MS		
Relapsing remitting (RRMS)	11	15
Secondary progressive (SPMS)	2	1
Primary progressive (PPMS)	3	0
Medications		
Dalfampridine^a^ use	1	2
Corticosteroids	0	0
Glatiramir	5	—
Interferon beta-1a	5	—
Natalizumab	2	—
Disease duration, years, mean (SD)	15.6 (11.5)	9 (4.3)
Length of follow-up, months, mean (SD)	41.2 (28)	39.8 (7)
Number of acute MS exacerbations^b^	2	4

^a^Indicated to help improve walking in adults with MS.

^b^Exacerbations calculated over length of follow-up period noted in table.
